# Supplementary material for: Carbohydrate Restriction in Type 1 Diabetes: A Realistic Therapy for Improved Glycaemic Control and Athletic Performance?
Source: Nutrients. 2019 May 7;11(5):1022. doi: 10.3390/nu11051022 (PMC6566372; doi:10.3390/nu11051022)
Supplement: Supplementary file 1 [file nutrients-11-01022-s001.pdf]

Table S1. Example diets for active 30-year-old female.

**30 year old active female – 2,200 kcal**		
Very Low Carbohydrate Diet (20-50 g/d)<br><br>**≈ 37 g Net**	Low Carbohydrate Diet[< 130 g/d or < 26% of energy (143 g/d)]<br><br>**≈ 116 g Net**	ADA Guidelines [45-60% of energy (248-330 g/d)]<br><br>**≈ 270 g Net**
Breakfast:<br>• ½ avocado<br>• 2 lg scrambled eggs<br>• 1 t butter<br>• Coffee or tea with sweetener<br><br>AM Snack:<br>• 1/3 C mixed nuts<br><br>Lunch:<br>• Spinach Salad with:<br>2 C spinach, 2 T onion, 1/3 english cucumber, ½ C cheddar cheese, 2 T oil and vinegar salad dressing<br>• 3 oz sirloin steak<br>• 4 spears cooked asparagus<br>• 1 t butter<br><br>PM Snack:<br>• 2 med stalks celery<br>• 2 T cream cheese<br><br>Dinner:<br>• Spring mix salad with:<br>• 2 C spring mix,1/3 english cucumber, ¼ C olives, 2 slices crumbled bacon, 1 T sunflower seeds, 2 T parmesan cheese, 2 T oil and vinegar salad dressing<br>• 1 C cauliflower<br>• 2 T grated parmesan cheese (add to salad or cauliflower)<br>• 1 t butter<br>• 4 oz chicken breast	Breakfast:<br>• 3/4 C cooked oatmeal (1/3 C dry rolled oats)<br>• 1 cup 2% milk<br>• ¼ C mixed nuts<br>• Coffee or tea with sweetener<br><br>AM Snack:<br>• 1/2 C plain Greek yogurt (not low fat)<br>• ½ tsp honey<br>• 1 tbsp ground flaxseeds<br><br>Lunch<br>• Spinach salad with:<br>• 2 C spinach, ¼ english cucumber, 5 cherry tomatoes, 1 small carrot (14 cm), ½ avocado, 2 T oil and vinegar salad dressing<br>• 1/3 C cooked quinoa<br>• 4 oz grilled salmon<br>• 1 C strawberries<br><br>PM Snack:<br>• 2 med stalks celery<br>• 2 T peanut butter<br><br>Dinner<br>• Spring mix salad with:<br>• 2 C spring mix, ¼ red pepper, 1 T sunflower seeds, 1 T grated parmesan cheese, 2 T oil and vinegar salad dressing<br>• 1/3 C sweet potato<br>• 2 spears cooked broccoli	Breakfast:<br>• 1 C raisin bran cereal<br>• 1 C skim milk<br>• ½ C strawberries<br>• Coffee or tea with sweetener<br><br>AM Snack:<br>• 2 T granola<br>• 175 g fat free (w sucralose) yogurt<br><br>Lunch:<br>• Spring Salad with:<br>• 1 C spring mix, ½ C mushrooms, ½ small red pepper, 1 T oil and vinegar salad dressing<br>• ½ can flaked tuna<br>• 1 cup cooked quinoa<br>• 1 med apple<br>• 2 oatmeal cookies<br><br>PM Snack:<br>• ½ C blueberries<br>• 1 chocolate chip cookie<br>Dinner:<br>• 1 C pasta with ½ C meat sauce<br>• 2 C romaine lettuce<br>• 2 T croutons<br>• 1 T Caesar salad dressing<br>• 1 C skim milk<br>• ½ C frozen yogurt

Evening snack:<br><br>• ½ C creamed cottage cheese (4.5% MF)<br>• 3 slices dry salami	• 2 t butter<br>• 2 T grated parmesan cheese (add to salad or broccoli)<br>• 4 oz chicken breast<br>• 1 medium apple	Evening Snack:<br><br>• 1 slice whole grain toast<br>• 1 T peanut butter<br>• ½ banana

Table S2. Example diets for active 30-year-old male.

**30 year old active male ≈ 2,500 kcal**		
Very Low Carbohydrate Diet (20-50 g/d)<br><br>**≈ 39 g net carb**	Low Carbohydrate Diet [< 130 g/d or < 26% of energy (143 g/d)]<br><br>**≈ 123 g net carb**	ADA Guidelines [45-60% of energy (248-330 g/d)]<br><br>**≈ 275 g net carb**
Breakfast:<br><br>• 1/2 avocado<br>• 2 lg scrambled eggs<br>• 1 t butter<br>• 2 oz smoked salmon<br>• Coffee or tea with sweetener<br><br>AM Snack:<br><br>• 1/2 C mixed nuts<br><br>Lunch:<br><br>• Spinach Salad wi<br>• 2 C spinach, ½ avocado, 1/3 english cucumber, 2 T diced onion, 2 T sunflower seeds, ½ C cheddar cheese, 2 T oil and vinegar salad dressing<br>• 3 oz top sirloin steak<br>• 4 spears asparagus<br>• 1 t butter<br><br>PM Snack:<br><br>• 2 med stalks celery<br>• 2 T cream cheese<br><br>Dinner:<br><br>• Spring Salad with:	Breakfast:<br><br>• ¾ C cooked oatmeal (1/3 C dry oats)<br>• 1 C 2% MF milk<br>• ½ C mixed nuts<br>• Coffee or tea with sweetener<br><br>AM Snack:<br><br>• ½ C plain Greek yogurt (not low fat)<br>• ½ tsp honey<br>• 1 T ground flaxseeds<br><br>Lunch:<br><br>• Spinach Salad with: 2 C spinach, ¼ English cucumber, 1 small carrot (14 cm), 5 cherry tomatoes, ½ avocado, 2 T oil and vinegar salad dressing<br>• 1/3 C cooked quinoa<br>• 4 oz grilled salmon<br>• 1 C strawberries<br><br>PM Snack:<br><br>• 2 med stalks celery<br>• 2 T peanut butter<br><br>Dinner:<br><br>Spring Salad with:	Breakfast:<br><br>• 1 ½ C raisin bran<br>• 1 C skim milk<br>• ½ C strawberries<br>• Coffee or tea with sweetener<br><br>AM Snack:<br><br>• ¼ C almonds<br>• 2 T granola<br>• 175 g fat free (w sucralose) yogurt<br><br>Lunch:<br><br>• Spring Salad with: 1 C spring mix, ½ small red pepper, ½ C mushrooms, ½ avocado, 2 T oil and vinegar salad dressing<br>• 1 C cooked quinoa<br>• ½ can flaked tuna<br>• 1 med apple<br>• 2 oatmeal cookies<br><br>Dinner:<br><br>• 1 C cooked pasta with ½ C meat sauce<br>• 2 C romaine lettuce, ¼ C croutons, 1 T Caesar salad

• 2 C spring mix, ¼ C olives, 2 slices crumbled bacon, 1 T sunflower seeds, 2 T oil and vinegar salad dressing<br>• 1 C cauliflower<br>• 2 T grated parmesan cheese (add to salad or cauliflower)<br>• 1 t butter<br>• 4 oz chicken breast<br><br>Evening Snack:<br><br>• 3 slices hard salami	• 2 C spring mix, ¼ red pepper diced, 2 T sunflower seeds, 2 T oil and vinegar salad dressing<br>• 1/3 C sweet potato<br>• 2 spears steamed broccoli<br>• 2 t butter<br>• 2 T parmesan cheese (add to salad or broccoli)<br>4 oz chicken breast	dressing<br>• 1 C skim milk<br>• ½ C frozen yogurt<br><br>Evening Snack:<br><br>• 1 slice whole grain toast<br>• 1 T peanut butter<br>• ½ banana

†Harris-Benedict Equation for Estimating Energy Requirement:
30 year old active male: 66.5 + 13.8 (weight) + 5 (height) − 6.8 (age)
= 66.5 + 13.8(70) + 5(174) – 6.8(30)
= 66.5 + 966 + 870 – 204
= 1698.5 X Activity Factor of 1.5 = 2,550 kcal
30 year old active female: 655 + 9.6 (weight) + 1.8 (height) − 4.7 (age)
= 655 + 9.6(60) + 1.8(161) – 4.7 (30)
= 655 + 576 + 290 – 141
= 1480 X Activity Factor of 1.5 = 2,220 kcal
Men – average height in Canada = 174 cm
Women average height in Canada = 161cm
Assuming BMI of 23, average weight of male = 70 kg, average weight of female = 60 kg
T = tablespoon; t = teaspoon; C = cup
